# Systematic review of the diagnostic accuracy of noninvasive intracranial pressure monitoring using optic nerve sheath diameter and the Brain4Care system

**DOI:** 10.1016/j.bas.2026.106036

**Published:** 2026-04-06

**Authors:** Marcus Roland Victor Gustafsson, Laura Lippa, Grace Barros de Sá, Fartein Velle, Elham Rostami

**Affiliations:** a Department of Physiology and Pharmacology, Karolinska Institutet, Stockholm, Sweden; b Department of Medical Sciences, Section of Neurosurgery, Uppsala University, Uppsala, Sweden; c Unit of Neurosurgery, Department of Neurosciences, Azienda Ospedaliera Universitaria Senese Le Scotte, Siena, Italy; d Universidade Do Estado Do Rio de Janeiro (UERJ), Rio de Janeiro, RJ, Brazil

**Keywords:** Intracranial pressure, Noninvasive monitoring, Optic nerve sheath diameter, Brain4Care, Ultrasound, Neuromonitoring, Cerebrospinal fluid pressure, Intracranial hypertension, Intracranial compliance

## Abstract

**Background:**

Assessing the intracranial pressure (ICP) with accuracy is essential in management of acute brain injury. Invasive monitoring remains the gold standard but carries procedural risks and requires neurosurgical competence. Noninvasive methods such as optic nerve sheath diameter (ONSD) ultrasonography and the Brain4Care (B4C) system have emerged as potential alternatives for estimating ICP or intracranial compliance.

**Methods:**

A systematic search of PubMed and Cochrane Library was conducted for studies published up to October 2025 evaluating ONSD or B4C against invasive ICP monitoring in adult patients. Data on diagnostic performance, including sensitivity, specificity and correlation with invasive ICP, were extracted and summarized.

**Results:**

A total of 47 studies was included, comprising 36 ONSD and 11 B4C investigations. ONSD cut-offs ranged from 4.1 to 7.2 mm, most commonly between 5.0 and 5.9 mm. Reported sensitivities were 70-100%, specificities 70-100% and correlation coefficients r = 0.64-0.90 with invasive ICP. B4C studies evaluated the P2/P1 ratio and time-to-peak (TTP) parameters, with sensitivities of 70-100% and specificities 41-92%. While some studies demonstrated strong correlations with invasive ICP (r up to 0.98), others reported modest accuracy and low specificity, emphasizing dependence on waveform quality and patient population.

**Conclusion:**

ONSD currently remains as the most validated noninvasive method for estimating raised ICP, demonstrating consistently high diagnostic accuracy across studies. The B4C system shows promise for continuous, noninvasive monitoring of intracranial compliance but requires further validation in larger, multicenter settings.

## Highlights

• ONSD shows consistently high diagnostic accuracy for elevated ICP.
• Brain4Care demonstrates high sensitivity but variable specificity.
• ONSD is the most validated noninvasive ICP screening method.
• Brain4Care enables continuous monitoring of intracranial compliance.
• Further multicenter validation is required for Brain4Care.

## 1 Introduction

Intracranial pressure is a critical determinant of poor neurological outcomes in patients with traumatic brain injury, subarachnoid hemorrhage and other acute cerebral pathologies. Therefore, monitoring of ICP remains essential to guide therapeutic interventions and prevent cerebral ischemia or herniation. As of now, the gold standard involves invasive monitoring using intraventricular or intraparenchymal catheters, which provides direct pressure measurements but carries risks of infection, hemorrhage and malfunction and requires neurosurgical expertise. These limitations have fueled a growing interest in reliable noninvasive neuromonitoring methods that can estimate ICP or intracranial compliance accurately (Sallam et al., 2021; Brasil et al., 2024a; Robba et al., 2018)

Several noninvasive neuromonitoring modalities have been developed to overcome the limitations of invasive ICP measurement. These include ultrasound estimation of the optic nerve sheath diameter (ONSD), transcranial doppler (TCD), near-infrared spectroscopy (NIRS) and more recently the Brain4Care (B4C) system by assessing cranial compliance (Sallam et al., 2021; Brasil et al., 2024a). ONSD exploits the anatomical continuity between the subarachnoid space and the optic nerve sheath, where increased ICP leads to sheath distension which is measurable by ultrasound. This method has been investigated in previous meta-analyses, demonstrating good diagnostic accuracy for detecting intracranial hypertension, though methodological heterogeneity and operator dependency limit its widespread adoption (Robba et al., 2018). In contrast, the B4C system represents an approach that measures cranial deformations related to the cardiac-induced ICP pulse waveform. By analyzing the shape and timing of the P1 and P2 waveform peaks, the system estimates intracranial compliance and indirectly reflects ICP noninvasively (Brasil et al., 2024a). Preliminary studies show that B4C measurements correlate with invasive ICP values, but the available evidence is limited and varies between clinical settings.

Previous studies have validated the potential of the B4C system for noninvasive ICP assessment. In a prospective study of stroke patients, de Moraes et al. demonstrated a strong correlation between B4C-derived waveform parameters and invasive ICP monitoring, with high sensitivity for detecting intracranial hypertension using the P2/P1 ratio and time-to-peak (TTP) metrics (de Moraes et al., 2022a). These findings suggest that waveform morphology can reflect intracranial compliance changes that precede ICP elevation. Frigieri et al. found that a P2/P1 ratio ≤0.8 effectively ruled out intracranial hypertension (sensitivity 92%), whereas values ≥ 1.4 and TTP ≥0.3 s showed high specificity (90-92%) for confirming raised ICP (Frigieri et al., 2025). However, de Moraes et al. reported substantially lower specificity for B4C parameters, only 45-50% for both P2/P1 and TTP, despite high sensitivity (100% and 86%, respectively). Their study concluded that while B4C measurements correlated with invasive ICP (r = 0.35) and that the technique's diagnostic accuracy was modest and highly dependent on waveform quality and clinical context (de Moraes et al., 2023).

Even though interest in noninvasive neuromonitoring is growing, current evidence remains fragmented, with variable performance across techniques and patient populations (Sallam et al., 2021). Among the available modalities, ONSD is one of the most extensively studied and validated approaches for estimating intracranial pressure noninvasively, while the B4C system represents a newer technique. Therefore, we conducted a systematic review focusing on these two methods to evaluate their diagnostic accuracy and clinical applicability compared with invasive ICP monitoring in adult patients.

## 2 Methods

### 2.1 Overview

The objective was to evaluate the diagnostic accuracy and clinical applicability of two noninvasive neuromonitoring methods, ONSD ultrasonography and the B4C system compared with invasive ICP monitoring in adult patients.

### 2.2 Search strategy

A systematic search of the PubMed database and Cochrane Library was performed to identify studies evaluating noninvasive ICP monitoring methods, specifically ONSD ultrasonography and the B4C system compared with invasive ICP measurements. The search was conducted using predefined search strings and limited to English-language studies involving human adults published between January 2008 and October 2025.

The search strategies were as follows:

ONSD: (“optic nerve sheath diameter” OR ONSD OR “optic nerve ultrasonography”) AND (“intracranial pressure” OR ICP) AND (“noninvasive” OR “non-invasive”) AND (“accuracy” OR “diagnostic” OR “validation” OR “sensitivity” OR “specificity")

B4C: (“brain4care” OR “noninvasive intracranial compliance” OR “noninvasive intracranial pressure” OR ″B4C”) AND (“intracranial pressure” OR “ICP")

### 2.3 Eligibility criteria

Inclusion criteria:

1. Adult human population (≥18 years)
2. Noninvasive assessment of ICP using ONSD ultrasound or the B4C system Comparison against invasive ICP monitoring (ventricular, intraparenchymal or lumbar puncture)
3. Reported diagnostic accuracy metrics (ex. sensitivity, specificity, AUC, or correlation coefficients)

Exclusion criteria:

1. Pediatric, animal, or phantom studies
2. Studies using other noninvasive modalities (ex. TCD, NIRS, EEG)
3. Case reports with <3 patients
4. Studies without invasive ICP reference data.

### 2.4 Study selection

**Fig. 1:**
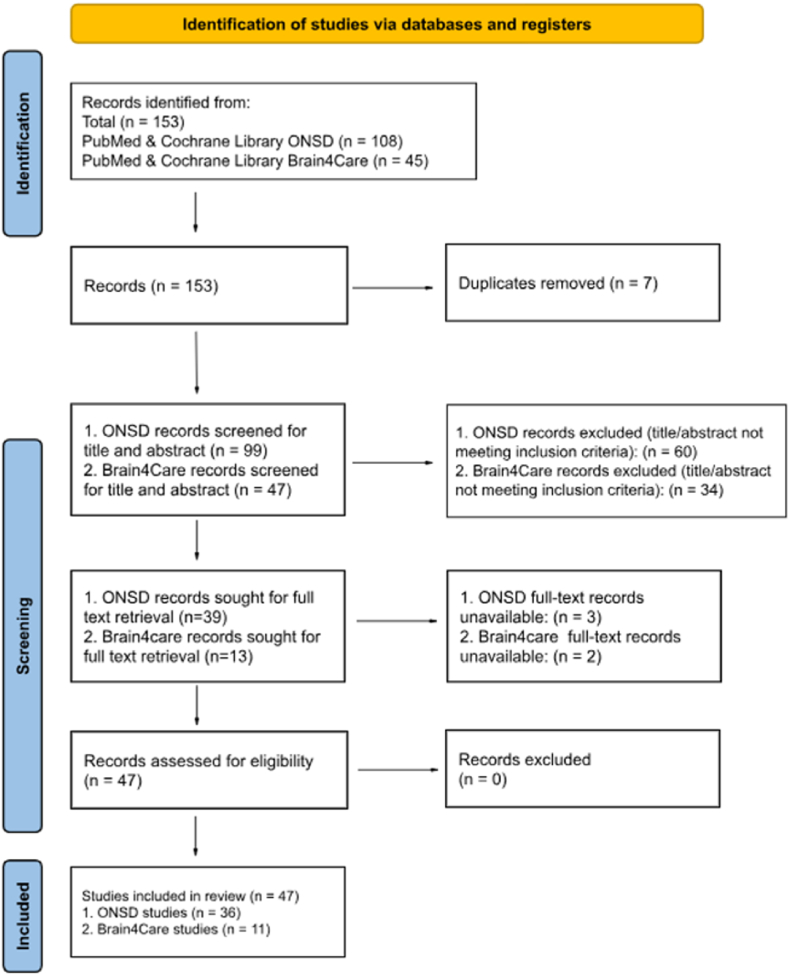
PRISMA-flowchart.

Two reviewers independently screened titles and abstracts for relevance. Full-text articles were retrieved for assessment. A total of 153 studies were identified: 108 ONSD and 45 B4C. After screening and full-text review, 36 ONSD and 11 Brain4Care studies met the inclusion criteria. A summary of the selection process is presented in the PRISMA 2020 flowchart (Fig. 1).

### 2.5 Data selection

The following variables were recorded for each study: Author, year of publication, study design, population (sample size and diagnosis), reference standard for invasive ICP, diagnostic thresholds (ex. ONSD cut-off, P2/P1 ratio, TTP), sensitivity, specificity, confidence intervals, correlation coefficients between noninvasive and invasive ICP.

### 2.6 Data synthesis

For ONSD the data were summarized visually using forest plots to display the sensitivity and specificity. Only studies that reported both sensitivity and specificity values were included in the forest plot analysis, as these parameters allowed for direct visual comparison of diagnostic accuracy. For B4C, the results were described narratively given the limited number of studies and differences in measurement protocols. All figures and analyses were generated using R (version 4.5.1; The R Foundation for Statistical Computing, Vienna, Austria).

## 3 Results

### 3.1 Study selection

A total of 153 records were identified through database searches, including 108 ONSD-related and 45 Brain4Care-related records from PubMed and the Cochrane Library. After removal of 7 duplicates, 146 records remained for screening. Title and abstract screening were performed for 99 ONSD and 47 Brain4Care records. During this stage, 60 ONSD and 34 Brain4Care records were excluded for not meeting the inclusion criteria. Full-text assessment was sought for 39 ONSD and 13 Brain4Care articles. Of these, 3 ONSD and 2 Brain4Care studies were unavailable in full text. The remaining 47 studies were assessed for eligibility, and no additional studies were excluded. In total, 47 studies were included in the systematic review, comprising 36 ONSD studies and 11 Brain4Care studies (Fig. 1). Study selection was performed independently by two reviewers using Covidence. Although inter-rater agreement statistics were not formally calculated, all conflicts were resolved by consensus.

### 3.2 Study characteristics

The included studies were published between 2008 and 2025, predominantly prospective observational or diagnostic accuracy designs. Patient populations included TBI, SAH, stroke and idiopathic intracranial hypertension (IIH). Most studies used intraventricular or intraparenchymal methods in invasive ICP measuring as reference standard, while 9 studies used lumbar puncture. ICP thresholds defining intracranial hypertension ranged from 15 to 25 mmHg. Detailed study characteristics and outcomes are presented in Table 1, Table 2 (B4C).

**Table 1:** Characteristics and diagnostic performance of ONSD studies for detecting elevated intracranial pressure. Summary of studies evaluating the relationship between ONSD and ICP. The table presents study characteristics, patient populations, reference ICP measurement methods, cut-off values, sensitivity, specificity, AUC, correlation coefficients, main findings and study designs.

Author (Year)	n	Population/Setting	Reference ICP method	ONSD Cut-off (mm)	Sensitivity (%)	Specificity (%)	AUC/Correlation	Main finding	Study type
Pansell et al. (2025) (Pansell et al., 2025)	213	Adult ICU patients with TBI, SAH, severe COVID-19.	Invasive ICP	6.9 for men and 6.5 for women	NR	NR	AUROC: NR (similar to invasive ICP for detection of ICH).	ONSD correlated well with invasive ICP; demonstrated similar diagnostic accuracy for detecting elevated ICP (CCC 0.65-0.70).	Post-hoc analysis
Fu et al. (2025) (Fu et al., 2025)	238	TBI patients	Invasive ICP	NR, 5.03 in ICH vs 4.59 in normal ICP.	84	92	AUC 0.92 (ICP ≥15 mmHg); 0.89 (ICP ≥20 mmHg)	ONSD with deep learning accurately detected high ICP and agreed well with invasive measurements.	Retrospective study
Ferreira et al. (2024) (Ferreira et al., 2025)	40	Severe TBI patients	Invasive ICP	Cut-off 6.18	77.8	81.8	ONSD correlated well with invasive ICP; demonstrated similar diagnostic accuracy for detecting elevated ICP (CCC 0.65 - 0.70).	ONSD showed weak correlation with ICP; 6.18 mm cut-off had moderate accuracy; suitable as a complementary tool.	Prospective observational study
Netteland et al. (2025) (Netteland et al., 2025)	20	Aneurysmal subarachnoid hemorrhage	Invasive ICP	Median ONSD 6.63 mm (high ICP ≥15 mmHg) vs 5.70 mm (normal ICP); no fixed cut-off defined	NR	NR	AUC = 0.84 (95% CI 0.65 -0.96); r = 0.43 (p = 0.03) overall; r = 0.55 (p = 0.01) in nonsurgical subgroup	Automated ONSD moderately correlated with ICP	Prospective observational study
Lioi et al. (2024) (Lioi et al., 2024)	20	Adults with severe traumatic brain injury	Invasive ICP	Cut-off: 5.8	92	80	AUC 0.98 (ICP ≥15 mmHg); 0.94 (ICP ≥20 mmHg); r = 0.90 (p < 0.001)	ONSD strongly correlated with invasive ICP; 5.8 mm cut-off accurately detected ICP ≥15 mmHg; useful as a noninvasive screening tool for intracranial hypertension	Prospective observational study
Netteland et al. (2024) (Netteland et al., 2024)	26	Adult traumatic brain injury	Invasive ICP	NR	NR	NR	ONSD AUC = 0.72 (95% CI 0.56 - 0.86); r = 0.45 (p < 0.01)	ONSD correlated moderately with ICP	Prospective observational study
de Moraes et al. (2023) (de Moraes et al., 2023)	18	Adults with hemorrhagic and ischemic stroke	Invasive ICP	5.2	71.4	70.4	AUC 0.69 (95% CI 0.62 - 0.78); r = 0.29 (p = 0.01)	ONSD weakly correlated with ICP; 5.2 mm cut-off had moderate accuracy	Prospective observational study
Wu et al. (2022) (Wu et al., 2022)	49	Adults with brain injury (SAH, stroke or TBI)	Invasive ICP	NR	NR	NR	r = 0.799 (p < 0.001, supine); r = 0.358 for positional change	ONSD strongly correlated with ICP at admission but not with dynamic ICP changes; not reliable for continuous monitoring	Prospective observational study
Agrawal et al. (2020) (Agrawal et al., 2020)	120	Severe TBI patients	Invasive ICP	7.2	82	79	AUC 0.81 (ICP >22 mmHg); r = 0.36 (p < 0.0001)	ONSD modestly correlated with ICP; 7.2 mm threshold showed moderate accuracy.	Prospective diagnostic study
Robba et al. (2020) (Robba et al., 2020)	100	Adults with TBI, SAH, or ICH	Invasive ICP	5.3	70	75	AUC 0.78 (95% CI 0.68 - 0.88); r = 0.54 (p < 0.001)	ONSD moderately correlated with ICP; 5.3 mm cut-off had acceptable accuracy	Prospective observational study
Zoerle et al. (2020) (Zoerle et al., 2020)	20	Subarachnoid hemorrhage patients	Invasive ICP	NR (mean ONSD ≈ 6.4 mm)	NR	NR	No correlation (R^2^ ≈ 0; p > 0.05)	ONSD not correlated with ICP	Prospective observational study
Klinzing et al. (2020) (Klinzing et al., 2020)	10	Traumatic brain injury patients	Invasive ICP	6.4	NR	NR	AUC 0.64; r = 0.25 (p = 0.32)	ONSD not significantly correlated with ICP	Prospective single-center study
Gupta and Pachisia (2019) (Gupta and Pachisia, 2019)	100	Adults undergoing lumbar puncture	Lumbar puncture	6.3	77.3	92.3	AUC 0.93 (95% CI 0.86 - 0.97); r = 0.715 (p < 0.001)	ONSD strongly correlated with CSF pressure; 6.3 mm cut-off accurately detected CSF pressure >20 cmH_2_O	Prospective observational study
Du et al. (2020) (Du et al., 2020)	49	TBI patients	Invasive ICP	Cut-off 5.53	80	79.3	AUC 0.87 (95% CI 0.80 - 0.91); r = 0.61 (p < 0.05).	ONSD moderately correlated with ICP; 5.53 mm cut-off moderately accurate.	Prospective observational study
Martin et al. (2019) (Martin et al., 2019)	54	TBI patients	Invasive ICP	5.6	100	NR	AUC 0.73 (95% CI 0.59 - 0.86)	ONSD higher in patients with early high ICP; 5.6 mm cut-off had perfect sensitivity but moderate accuracy.	Prospective observational study
Chen et al. (2019) (Chen et al., 2019)	84	Adults undergoing lumbar puncture for diagnostic purposes	Lumbar puncture	NR (ONSD range 3.57–5.63 mm; elevated ICP >200 mmH_2_O)	NR	NR	r = 0.482 (p < 0.01) before LP; r = 0.451 (p < 0.01) for ΔONSD - ΔICP	ONSD correlated moderately with ICP and decreased immediately after CSF pressure reduction	Prospective observational study
Kishk et al. (2018) (Kishk et al., 2018)	99	Adult females with idiopathic intracranial hypertension	Lumbar puncture	Cut-off: 6.05	73.2	91.4	AUC 0.85 (95% CI 0.81 - 0.89)	ONSD significantly larger in IIH vs controls; 6.05 mm cut-off showed good accuracy	Case-control study
Ebraheim et al. (2018) (Ebraheim et al., 2018)	24 patients + 30 controls	Adult females with idiopathic intracranial hypertension	Lumbar puncture	Cut-off: 6.2	87.5	100	AUC 0.98 (95% CI 0.94–1.01); r = 0.13 (p = 0.56)	ONSD significantly larger in IIH; 6.2 mm cut-off showed excellent diagnostic accuracy	Prospective case-control study
Wang et al. (2018) (Wang et al., 2018)	60	Adults with suspected elevated ICP of various causes	Lumbar puncture	NR	NR	NR	r = 0.798 (p < 0.001) baseline; r = 0.702 (p < 0.001) for change	ONSD strongly correlated with ICP and decreased after pressure reduction	Prospective observational study
Liu et al. (2017) (Liu et al., 2017)	110	Adults undergoing lumbar puncture for (meningitis, encephalitis, IIH, SAH, etc.)	Lumbar puncture	5.6	86.2	73.1	AUC 0.86; r = 0.61 (p < 0.001)	ONSD significantly correlated with ICP; 5.6 mm cut-off showed good diagnostic accuracy	Prospective observational study
Ganslandt et al. (2018) (Ganslandt et al., 2018)	14	Adults with TBI or SAH	Invasive ICP	NR (device-derived ICP ≥17 mmHg threshold)	75.4	88.9	AUC 0.895; r = 0.82 (p < 0.0001)	Strong correlation between noninvasive and invasive ICP for detecting ICP ≥17 mmHg	Prospective comparative study
Robba et al. (2017) (Robba et al., 2017)	64	Adults with TBI, SAH, or ICH	Invasive ICP	Cut-off: 6.25	NR	NR	AUC 0.91 (95% CI 0.88 - 0.95); r = 0.76 (p < 0.001)	ONSD strongly correlated with invasive ICP, identified raised ICP (≥20 mmHg) with high accuracy	Prospective observational study
Toscano et al. (2017) (Toscano et al., 2017)	21 patients + 31 controls	Acute brain injuries (ICH, SAH, trauma, tumor, postanoxic coma)	Invasive ICP	NR (mean ONSD pre-BD 5.4–5.5 mm; post-BD >7.0 mm)	NR	NR	r = 0.895 (p < 0.001)	ONSD strongly correlated with ICP	Retrospective observational study
Wang et al. (2017) (Wang et al., 2017)	315	Adults undergoing lumbar puncture for suspected elevated ICP	Lumbar puncture	NR	NR	NR	r = 0.76 (p < 0.001); ICC = 0.86 (95% CI 0.79 - 0.90)	ONSD strongly correlated with ICP	Cross-sectional observational study
Raffiz and Abdullah (2017) (Raffiz and Abdullah, 2017)	41	Adult, TBI and non-traumatic	Invasive ICP	Cut-off: 5.21	95.8	80.4	AUC 0.96 (95% CI 0.92 - 1.00); r = 0.82 (p < 0.01)	ONSD strongly correlated with ICP; 5.21 mm cut-off highly accurate for detecting ICP >20 mmHg; more accurate in TBI than non-traumatic cases	Prospective observational study
Vaiman et al. (2016) (Vaiman et al., 2016)	443	Adults with spontaneous ICH or SAH and non-traumatic	Invasive ICP	5.5	94	83	r = 0.82 (p < 0.05)	ONSD >5.5 mm accurately predicted elevated ICP; strong correlation with invasive ICP	Retrospective CT-based observational study
Wang et al. (2015) (Wang et al., 2015)	279	Adults with suspected elevated ICP undergoing lumbar puncture	Lumbar puncture	4.1	95	92	AUC 0.97 (95% CI 0.95 - 0.98)	ONSD strongly predicted elevated ICP; 4.1 mm cut-off showed high sensitivity and specificity	Cross-sectional study
Frumin et al. (2014) (Frumin et al., 2014)	27	Adults with various intracranial pathologies (TBI, SAH, ICH, hydrocephalus) in neuro-ICU, USA	Invasive ICP (external ventricular drain)	5.2	83.3	100	AUC 0.87 (95% CI 0.67 - 0.96); r = 0.41 (p = 0.03)	ONSD moderately correlated with ICP; 5.2 mm cut-off showed good sensitivity and perfect specificity for ICP >20 mmHg	Prospective observational study
Ragauskas et al. (2014) (Ragauskas et al., 2014)	92	Adults undergoing lumbar puncture	Lumbar puncture	5.0	37	58.5	AUC 0.57 (95% CI 0.47 - 0.67)	ONSD showed poor correlation and diagnostic accuracy for detecting ICP >14.7 mmHg	Prospective clinical study
Rajajee et al. (2011) (Rajajee et al., 2011)	65	Adults with acute brain injury (SAH, TBI, ICH, tumors, shunt malfunction)	Invasive ICP	4.8	96	94	AUC 0.98 (95% CI 0.96 - 0.99); r = 0.73 (p < 0.0001)	ONSD strongly correlated with ICP; 4.8 mm cut-off showed high accuracy for detecting ICP >20 mmHg	Prospective observational study
Moretti et al. (2009) (Moretti et al., 2009)	63	Adults with spontaneous intracerebral or subarachnoid hemorrhage	Invasive ICP	5.2	93.1	73.9	AUC 0.93 (95% CI 0.85 - 0.97); r = 0.70 (p < 0.0001)	ONSD strongly correlated with ICP; 5.2 mm cut-off accurately detected ICP >20 mmHg	Prospective observational study
Geeraerts et al. (2008) (Geeraerts et al., 2008)	37	Adults with severe TBI, SAH, hematoma, or stroke	Invasive ICP	Cut-off: 6.26	95	79	AUC 0.91 (95% CI 0.82 - 0.96); r = 0.71 (p < 0.0001)	ONSD strongly correlated with ICP; 5.86 mm cut-off accurately predicted ICP ≥20 mmHg; values < 5.8 mm effectively ruled out raised ICP	Prospective observational study
Soldatos et al. (2008) (Soldatos et al., 2008)	50 patients and 26 controls	Adults with traumatic brain injury	Invasive ICP	5.7	74.1	100	AUC 0.93 (95% CI 0.79 - 0.99); r = 0.68 (p = 0.002)	ONSD correlated with invasive ICP; 5.7 mm cut-off accurately detected raised ICP.	Prospective observational study
Kimberly et al. (2008) (Kimberly et al., 2008)	15	Adults with spontaneous or traumatic intracerebral hemorrhage	Invasive ICP	5.0	88	93	AUC 0.93 (95% CI 0.84 - 0.99); r = 0.59 (p < 0.0005)	ONSD significantly correlated with invasive ICP; 5.0 mm cut-off accurately detected ICP >20 cm H20	Prospective observational study
Cardim et al. (2019) (Cardim et al., 2019)	11	Adults with hypoxic ischemic brain injury after cardiac arrest, ICU, Canada	Invasive ICP	5,95	86	100	AUC 0.96 (95% CI 0.90 - 1.00); r = 0.53 (p < 0.0001)	ONSD moderately correlated with invasive ICP	Prospective observational study
Tian et al. (2024) (Tian et al., 2024)	50	ICU patients with acute brain injury	Invasive ICP	6,56	77.4	84.3	AUC 0.843; r = 0.50 - 0.61	ONSD moderately correlated with ICP	Prospective observational study

**Table 2:** Characteristics and diagnostic performance of B4C studies for noninvasive intracranial compliance and pressure assessment. Summary of studies evaluating the B4C system. The table outlines patient populations, reference methods, P2/P1 ratio, TTP, sensitivity, specificity, AUC or correlation values, principal findings and study design.

Author (Year)	n	Population	Reference method	Main B4C parameter	Cut-off (ratio and s)	Sensitivity%	Specificity%	AUC or Correlation	Main finding	Study type
Frigieri et al. (2025) (Frigieri et al., 2025)	124	Acute brain injury patients	Invasive ICP	P2/P1 ratio and TTP	P2/P1 = 1.4	11	90	NR	B4C shows good diagnostic accuracy for noninvasive IH detection.	Retrospective multicenter analysis
Uysal et al. (2024) (Uysal et al., 2025a)	21	Acute brain injury patients	Invasive ICP	P2/P1 ratio, TTP, and compliance	NR	NR	NR	R = 0.982 (95% CI 0.980 - 0.984, p < 0.001); CCC: log (TCD) = 0.76, P2/P1 = 0.42, TTP = 0.33	Supports feasibility of B4C for noninvasive compliance monitoring.	Retrospective single-center study
Brasil et al. (2024) (Brasil et al., 2024b)	98	Acute brain injury patients	Invasive ICP	P2/P1 ratio	1.13	100	1-5	AUC = 0.70 (mortality); AUC = 0.72 (ICP >20 mmHg with eICP + P2/P1)	Moderate correlation with invasive ICP, strong negative predictive value for ruling out IH.	Prospective observational study
de Moraes et al. (2024) (de Moraes et al., 2024)	69	Acute brain injury patients	Invasive ICP	P2/P1 ratio	1.13	93	60	AUC = 0.83, r = 0.44 (p < 0.001)	B4C emonstrated strong rule-out performance for IH.	Retrospective multicenter analysis
de Moraes et al. (2023) (de Moraes et al., 2023)	18	Acute brain injury patients	Invasive ICP	P2/P1 ratio and TTP	P2/P1 1.06 and TTP 0.20	P2/P1 = 100 85.7, TTP = 85.7	P2/P1 = 45.5, TTP = 50	AUC = 0.79 (P2/P1) 0.69 (TTP) r = 0.35 (p < 0.001)	P2/P1 and TTP moderately correlated with ICP.	Prospective study
Brasil et al. (2023) (Brasil et al., 2023)	72	Acute brain injury patients	Invasive ICP	P2/P1 ratio	>1.2	85	77	AUC 0.88 to predict IHT; AUC 0.71 for early death	P2/P1 > 1.2 correlated with IHT (ICP >20 mmHg) and early death, good diagnostic accuracy.	Prospective multicenter cross-sectional study
de Moraes et al. (2022) (de Moraes et al., 2022b)	18	Acute brain injury patients	Invasive ICP	P2/P1 ratio, TTP	P2/P1 1.06 and TTP 0.20	P2/P1 = 100, TTP = 85.7	P2/P1 = 45.4, TTP = 50	AUC = 0.79 (P2/P1), 0.69 (TTP); r = 0.43 (ICP correlation)	Detected all ICP >20 mmHg cases. Noninvasive waveform monitoring feasible for ICP screening.	Prospective study
Ballestero et al. (2023) (Ballestero et al., 2023)	22	Acute brain injury patients	Invasive ICP	P2/P1 ratio, TTP	P2/P1 ≥ 1.2–1.4; TTP ≥0.25–0.27 s	53.6–100	0–73.6	AUC = 0.484 - 0.497 (nICP); 0.828 (iICP)	B4C showed good waveform similarity but low specificity and accuracy for detecting intracranial hypertension.	Prospective study
Brasil et al. (2025) (Brasil et al., 2025)	71	Acute brain injury patients	Invasive ICP	P2/P1 ratio	Cut-off not defined	NR	NR	r = 0.88 (p < 0.001)	Demonstrates feasibility of fully noninvasive CSC estimation.	Prospective study
Hassett et al. (2023) (Hassett et al., 2023)	24	Acute brain injury patients	Invasive ICP	nPAx	NR	NR	NR	Repeated measures correlation r = 0.70 (95% CI 0.687 - 0.717, p < 0.0005)	Strong correlation and excellent agreement between noninvasive nPAx and invasive PAx.	Retrospective study
Uysal et al. (2025) (Uysal et al., 2025b)	21	Acute brain injury patients	Invasive ICP	P2/P1 ratio, TTP	NR	NR	NR	Repeated measures correlation: r = 0.982 (95% CI 0.980 - 0.984, p < 0.001).	Demonstrates B4C feasibility for noninvasive compliance monitoring.	Retrospective study

### 3.3 Optic Nerve Sheath Diameter (ONSD)

**Fig. 2:**
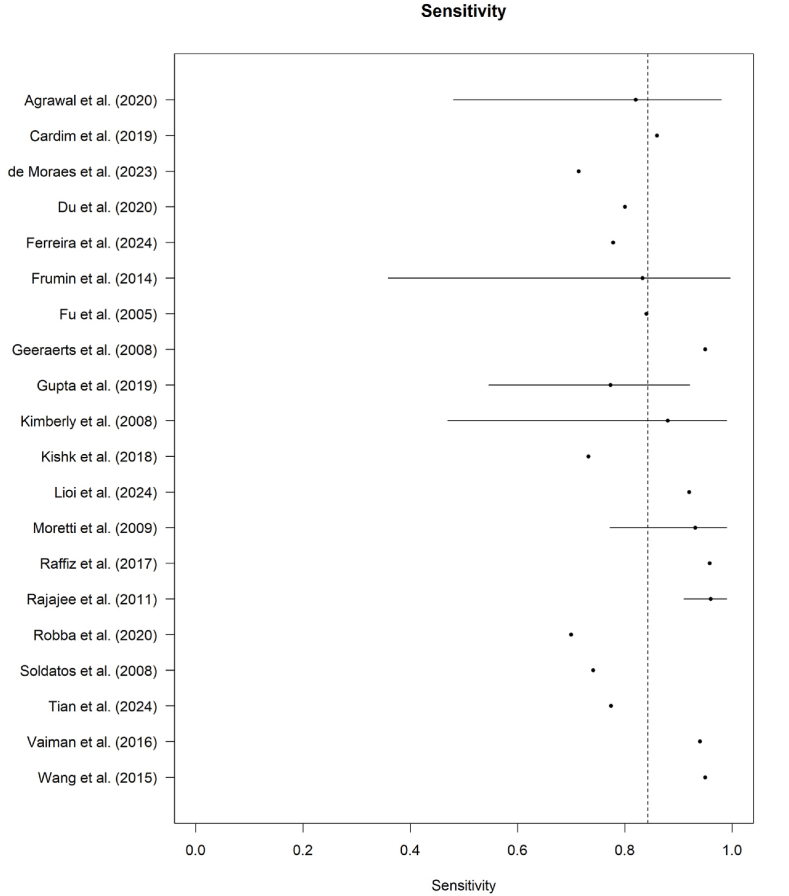
Forest plot of sensitivity estimates for ONSD in detecting elevated intracranial pressure.

**Fig. 3:**
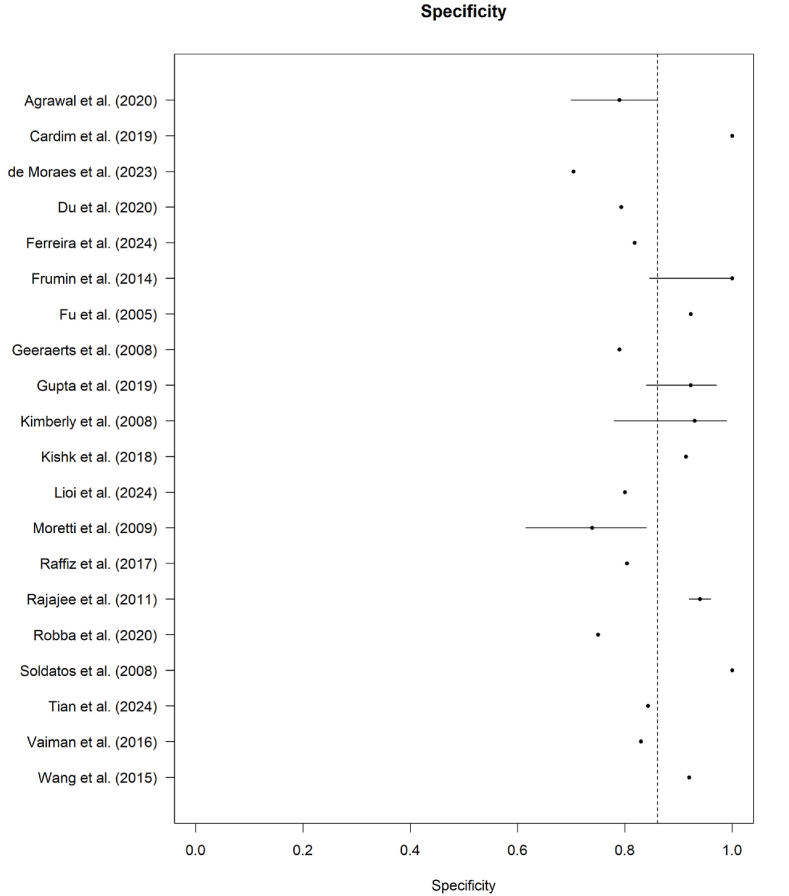
Forest plot of specificity estimates for ONSD in detecting elevated intracranial pressure.

**Fig. 4:**
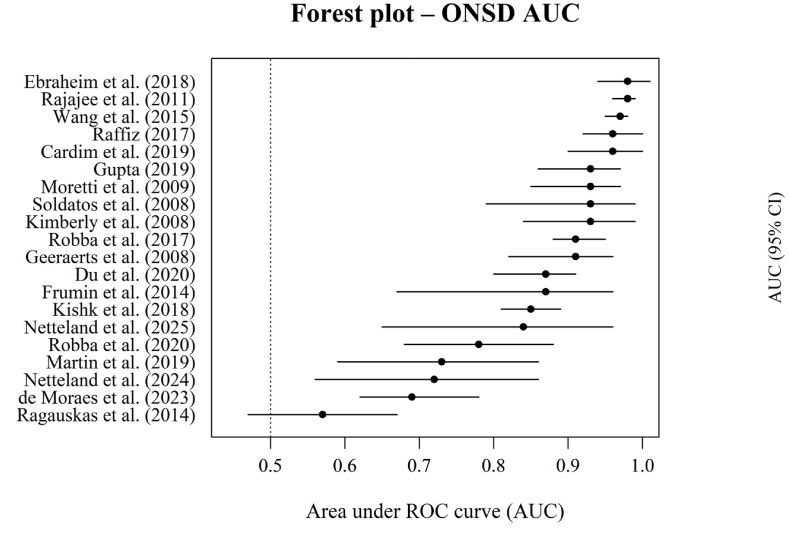
Forest plot of AUC for ONSD in detecting elevated intracranial pressure.

**Fig. 5:**
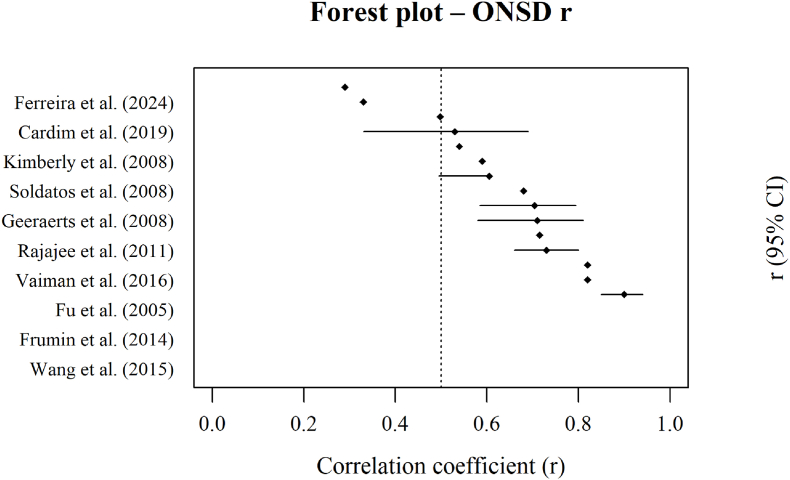
Forest plot of correlation coefficients between ONSD and invasive ICP measurements.

Out of the 36 studies which have investigated the diagnostic performance of ONSD ultrasonography for detecting elevated ICP, ONSD cut-offs varied between 4.1 and 7.2 mm, most frequently within 5.0-5.9 mm. Sensitivities ranged from 70% to 100% and specificities from 70% to 100%, with most AUC values were above 0.80, although several studies reported more modest discriminatory performance, most notably Ragauskas et al. with a sensitivity of 37% and a specificity of 58.5%. Several studies reported strong correlation between ONSD and invasive ICP measurements, with correlation coefficients ranged from weak to strong (approximately r = 0.25-0.90), with most studies reporting moderate to strong associations (see Fig. 5). (ex. Lioi et al., 2024; Raffiz and Abdullah, 2017; Geeraerts et al., 2008). Studies reporting weaker correlations (ex. Ferreira et al., 2024; de Moraes et al., 2023; Klinzing et al., 2020) may reflect small sample sizes or patient heterogeneity. Visual inspection of the forest plots, Fig. 2, Fig. 3, indicates that most ONSD studies reported high diagnostic accuracy, particularly in patients with traumatic brain injury and subarachnoid hemorrhage (see Fig. 4).

### 3.4 Brain4Care (B4C)

Of the 11 studies published between 2022 and 2025 evaluating the B4C system for ICP estimation or intracranial compliance. All compared P2/P1 ratio and TTP with invasive ICP monitoring. Cut-off values for intracranial hypertension ranged between P2/P1 = 1.06-1.4 and TTP = 0.20-0.30 s. Sensitivities ranged from 70% to 100% and specificities showed substantial variability, ranging from very low values in some cohorts to over 90% in others. In the largest multicenter study (Frigieri et al., 2025; n = 124), specificities reached 90-92% for higher thresholds (P2/P1 ≥ 1.4, TTP ≥0.3 s), whereas de Moraes et al. (2023) reported specificities of only 45 to 50% despite perfect sensitivity. Similarly, Brasil et al. (2024) found that the P2/P1 parameter had a 100% sensitivity but extremely low specificity 1-5% for detecting intracranial hypertension. Correlation coefficients between B4C and invasive ICP ranged from r = 0.35 to 0.98, with the highest correlations reported in controlled waveform recordings (Uysal et al., 2024). Overall, previous studies states that the B4C system shows potential for early detection of intracranial hypertension, though performance varies considerably depending on waveform quality and patient population.

The forest plot is displaying the sensitivity estimates of ONSD for detecting elevated intracranial pressure across included studies. Each point represents the reported sensitivity with corresponding confidence intervals, while the dashed vertical line indicates the pooled sensitivity estimate.

Forest plot illustrating the specificity of ONSD for detecting elevated intracranial pressure across included studies. Each point represents the reported specificity with corresponding confidence intervals, while the dashed vertical line indicates the pooled specificity estimate.

Forest plot illustrating the reported AUC values for ONSD ultrasonography in detecting elevated intracranial pressure across included studies. Each point represents the study-specific AUC with corresponding 95% confidence intervals, reflecting the discriminatory ability of ONSD compared with invasive ICP monitoring. The dashed vertical line indicates an AUC of 0.5, corresponding to no discriminative performance.

Forest plot showing the correlation coefficients between ONSD measurements and invasive ICP across included studies. Each point represents the reported correlation coefficient with corresponding 95% confidence intervals. The dashed vertical line indicates a moderate correlation r = 0.5, illustrating variability in the strength of association between ONSD and invasive ICP across study populations.

## 4 Discussion

This systematic review evaluated ONSD ultrasonography and the B4C system and how it correlates against invasive ICP monitoring. Out of the 36 ONSD and 11 B4C studies, both modalities showed diagnostic potential, though the evidence is at different stages. ONSD reliably demonstrated strong diagnostic accuracy, while B4C reflected intracranial compliance yet performed inconsistently among patient groups.

Our findings are consistent with previous meta-analyses indicating that ONSD serves as a robust and reproducible surrogate marker for elevated ICP (Robba et al., 2018; Dubourg et al., 2011; Rajajee et al., 2011). Several prospective studies confirmed strong correlations between ONSD and invasive ICP with pooled sensitivities and specificities typically above 85% (Raffiz and Abdullah, 2017; Geeraerts et al., 2008; Kimberly et al., 2008). Variability in optimal cut-off values, from 4.8 to 6.2 mm, likely reflects technical and operator-related differences, as previously discussed by Robba et al. and Ragauskas et al. (Robba et al., 2018; Ragauskas et al., 2014) These results support that ONSD remains the most validated noninvasive indicator of raised ICP.

In contrast, the B4C system represents a newer method that quantifies cranial deformation to derive waveform parameters such as the P2/P1 ratio and TTP. Early studies have demonstrated moderate correlations with invasive ICP (Frigieri et al., 2025; Uysal et al., 2025a). Frigieri et al. reported sensitivities up to 92% and specificities approaching 90-92% for higher cut-offs, whereas de Moraes et al. observed lower specificity 45-50% despite perfect sensitivity and Brasil et al. noted specificities as low as 1-5% (Frigieri et al., 2025; de Moraes et al., 2023; Brasil et al., 2024b). These differences suggest that waveform quality and sensor stability influence diagnostic performance.

In addition to ONSD and B4C, other ultrasound-based methods for ICP measurement are emerging such as the transcranial transmission ultrasound method (TTUS) which combines machine learning to exclude intracranial hypertension. In a study conducted by Krieg et al. (2024) TTUS achieved a sensitivity of 100% and a negative predictive value of 100% for detecting ICP >15 mmHg, on the other hand a specificity of 47%. Similar to the B4C system the TTUS method reflects changes in intracranial compliance and waveform morphology rather than measuring absolute ICP values.

When comparing B4C and ONSD [Table 1, Table 2], the latter shows more consistent diagnostic accuracy across patient populations, with sensitivities and specificities frequently between 80 and 95% and correlation coefficients typically above r = 0.6-0.9. In contrast, B4C studies showed greater variability, with sensitivities ranging from 70 to 100% and specificities reported as low as 1-50% in some cohorts. ONSD allows for fast assessment of ICP elevation, whereas B4C provides continuous evaluation of compliance. Overall, these findings indicate that ONSD is the more validated diagnostic tool at present.

Both ONSD and B4C have distinct clinical advantages. ONSD offers fast bedside estimation of ICH which is particularly useful in emergency and resource-limited settings (Robba et al., 2018; Geeraerts et al., 2008). Unlike ONSD, B4C continuously measures intracranial compliance and might identify changes earlier than visible ICP increases (Frigieri et al., 2025). Combining these approaches could improve early diagnosis and guide further care.

### 4.1 Limitations

This review has several limitations. There was methodological variation among the studies in terms of reference standards, measurement techniques and patient selection. Confidence intervals, sensitivity, specificity and correlation were inconsistently reported, and most studies were single-center with small cohorts. Although a meta-analysis was not performed, limiting quantitative comparison, the structured qualitative review provides a balanced summary of current evidence.

### 4.2 Future perspectives

Future studies should focus on standardizing ONSD measurements, including probe depth, imaging axis and averaging methods and on testing the B4C system in larger, multicenter patient groups. Instead of just confirming that B4C works in one small study or single hospital, researchers should test it in multiple settings to confirm that the results are reliable, reproducible and generalizable. Integrating these techniques with other noninvasive methods such as TCD and NIRS may further improve diagnostic accuracy and clinical applicability (Battaglini et al., 2022; Agarwal and Benedetti, 2024).

## 5 Conclusion

In conclusion, ONSD is currently the most validated noninvasive method for detecting raised ICP, while the B4C system shows promise for continuous monitoring of intracranial compliance. Both may play complementary roles in developing safer and more accessible neuromonitoring.

## Author contributions

Marcus Roland Victor Gustafsson and Grace Barros de Sá; drafted the main manuscript. Marcus Roland Victor Gustafsson performed the data extraction, statistical analysis, and data interpretation. Laura Lippa contributed to the study methodology. Elham Rostami and Fartein Velle supervised the project, provided critical revisions, and ensured methodological accuracy. All authors reviewed and approved the final manuscript.

## Ethics approval

Ethical approval was not required for this study, as it is a systematic review based exclusively on previously published data.

## Funding

No funding was received for this study.

## Declaration of competing interest

The authors declare no conflicts of interest.

## Data Availability

All data analyzed in this study are derived from published articles included in the systematic review. No new datasets were generated or analyzed.
